# The Significance and Process of Inflammation Involving Eicosapentaenoic and Docosahexaenoic Derivatives in Hashimoto’s Disease

**DOI:** 10.3390/nu17101715

**Published:** 2025-05-19

**Authors:** Małgorzata Szczuko, Klaudia Zawadzka, Urszula Szczuko, Leon Rudak, Jakub Pobłocki

**Affiliations:** 1 Department of Human Nutrition and Metabolomics, Pomeranian Medical University in Szczecin, 70-252 Szczecin, Polandleon.rudak88@gmail.com (L.R.); 2 Department of Endocrinology, Metabolic Diseases and Internal Diseases, Pomeranian Medical University in Szczecin, 70-252 Szczecin, Poland; jakub.poblocki@pum.edu.pl

**Keywords:** Hashimoto’s thyroiditis, omega-3 fatty acids, maresins, resolvins, protectins, inflammatory state, EPA, DHA

## Abstract

**Background:** The anti-inflammatory effects of omega-3 fatty acids and their derivatives are of considerable interest as a potential therapeutic agent in many diseases characterized by inflammation. **Methods:** This study aimed to measure the concentration of mediators derived from eicosapentaenoic (EPA) and docosahexaenoic (DHA) fatty acids. We included 33 women suffering from Hashimoto’s disease, with an average age of 37.58 ± 8.41 kg, in the study. The levels of EPA and DHA acids were examined using gas chromatography, and their derivatives were studied with liquid chromatography (HPLC). Patients were assessed after being put on a healthy and balanced diet supplemented with omega-3 fatty acids. **Results:** The results showed statistically significant correlations between the C-reactive protein (CRP) level and derivatives: resolvins E1 and D1 (RvE1, RvD1), 10S17R DiHDHA (Protectin DX), and 18RS HEPE (18-hydro(peroxy)-eicosapentaenoic acid) following the diet. There was also a significant correlation observed between Maresin 1 and free thyroxine (fT4). Moreover, a dependency between the RvD1 level and some anthropometric parameters was observed. **Conclusions:** The findings suggest that the chronic inflammatory state occurring in the course of Hashimoto’s thyroiditis (HT) is associated with increased synthesis of anti-inflammatory mediators of omega-3 fatty acids, particularly DHA derivatives. Consequently, these may affect the level of thyroid hormone synthesis, which should be considered in future research on biological drugs in Hashimoto’s therapy.

## 1. Introduction

Hashimoto’s disease affects individuals of all ages, including children, but it is most commonly diagnosed in people aged 45–65 years. The disease is diagnosed in both women and men; however, studies suggest that the incidence among women may be 10–20 times higher [1]. This discrepancy may be associated with the higher production of estrogen in the female body, which plays a role in the pathogenesis of the disease. Although environmental factors conducive to the occurrence of Hashimoto’s thyroiditis (HT) are known, the pathogenesis remains not fully understood [2]. It is likely caused by genetic predispositions, especially polymorphism in the major histocompatibility complex (HLA) and the cytotoxic T lymphocyte antigen 4 gene (CTLA-4), reduced function of regulatory T lymphocytes, and environmental factors (infections, cytokine therapies, selenium and iodine intake, stress). These lead to abnormalities in immunological tolerance to one’s own cells, resulting in autoimmune aggression against thyroid cells [3]. Hashimoto’s disease leads to the gradual destruction of the thyroid gland. This results in a decrease in thyroid hormone levels, leading to the development of hypothyroidism and symptoms such as chronic fatigue, dry skin, depression, metabolic disorders, and weight gain [4]. In the progression of HT, autoreactive lymphocytes and autoantibodies against thyroid peroxidase (ATPO) and thyroglobulin (ATG) infiltrate the thyroid tissue [3]. This process leads to an increased secretion of pro-inflammatory cytokines like interleukin 2 and 6 (IL-2, IL-6), interferon gamma, and tumor necrosis factor-alpha (TNF-α), and a reduction in the secretion of interleukin 4, 5, and 10 (IL-4, IL-5, IL-10), which are cytokines responsible for immunotolerance. Moreover, the aforementioned ATPO and ATG antibodies, produced by B lymphocytes, become cytotoxic or activate cytotoxic T lymphocytes. As a result, there is an increased expression of apoptotic factors and consequently increased apoptosis of thyrocytes, or follicular thyroid cells, progressively reducing hormone synthesis.

Essential fatty acids (EFAs) are a group of fats that the human body cannot produce, yet they are vital for proper body functioning. These include two groups of fatty acids, omega-3 and omega-6. The representative acid of the omega-3 family is alpha-linolenic acid (ALA), naturally found in products like flax seeds, walnuts, and chia. ALA can be metabolized into long-chain fatty acids—eicosapentaenoic acid (EPA) and docosahexaenoic acid (DHA). However, this synthesis is limited and insufficient. Therefore, EPA and DHA should be directly obtained from food or supplements [5]. EPA and DHA are primarily found in fish and their products (caviar, fish oil), especially fatty ones [6]. Both omega-3 and omega-6 are precursors of various lipid mediators involved in the inflammatory process, synthesized by cyclooxygenases or lipoxygenases. In the case of omega-6 acids, these are primarily pro-inflammatory eicosanoids such as prostaglandins, thromboxanes, or leukotrienes [7]. Anti-inflammatory metabolites of omega-3 include resolvins, maresins, and protectins [8]. Omega-6 and omega-3 fatty acids and their metabolites have opposite effects, so maintaining their appropriate proportion in the diet is essential for homeostasis [9].

Resolvins are anti-inflammatory mediators that can be divided into resolvin series E (RvE) and resolvin series D (RvD), derived from EPA and DHA, respectively [10]. The conversion of EPA to 18R-HEPE can lead to the synthesis of series E resolvins, facilitated by endothelial cells with the involvement of COX-2. This process is significantly more efficient with the additional participation of acetylsalicylic acid (ASA). The 18-hydro(peroxy)-eicosapentaenoic acid (18R-HEPE) can also be released to neighboring leukocytes where with the involvement of 5-LOX, it is converted to series 1 and 2 resolvins [11]. Regarding series D resolvins, they are metabolites formed from DHA. We can divide this series of resolvins into two groups. The formation of the first group requires acetylsalicylic acid, which participates in the acetylation of COX-2. The reaction involving it results in the formation of AT-RvD1, AT-RvD2, AT-RvD3, and AT-RvD4 [12]. Numerous studies have already shown the role of resolvins in actively reducing inflammation. These mediators lower the levels of adhesive leukocytes (CD11/CD18) and also influence the reduction in pro-inflammatory interleukin synthesis (especially IL-12 and IL-6) and tumor necrosis factor-alpha (TNF-α) [13]. It has also been shown that the presence of resolvins enhances the apoptosis of polymorphonuclear neutrophils (PMN) [14]. The dampening of inflammation by resolvins also involves increasing phagocytosis and promoting the secretion of anti-inflammatory cytokines such as interleukin-10 (IL-10) [15].

Maresins (MaR) are the least known group of anti-inflammatory mediators to date. It is suggested that their synthesis only occurs during the resolution phase of inflammation [16]. Their formation occurs in macrophages from DHA with the involvement of 12-lipoxygenase (12-LOX). Maresin biosynthesis involves the oxidation of DHA and then the epoxidation of the resulting intermediate. This leads to the formation of 13S,14S-epoxy-maresins, which are then converted to maresins [17]. Maresins primarily inhibit the migration of neutrophils. Additionally, like resolvins, they suppress the production of inflammatory cytokines (especially IL-1β, IL-6, or TNF-α) and increase the production of the anti-inflammatory cytokine IL-10 [17]. They also reduce the activity of cells such as CD4+, CD8+, or Th17. The anti-inflammatory action of maresins also involves increasing the induction of Treg cells, a narrow population of T lymphocytes that inhibit the response of effector lymphocytes against self-antigens, thereby preventing the development of autoimmune disease [18]. One of the most important receptors of Treg cells that “turns off” other cells, CTLA-4, is currently being produced as a biological drug—abatacept or belatacept. These drugs are currently used in the treatment of rheumatoid arthritis [19].

**Figure 1:**
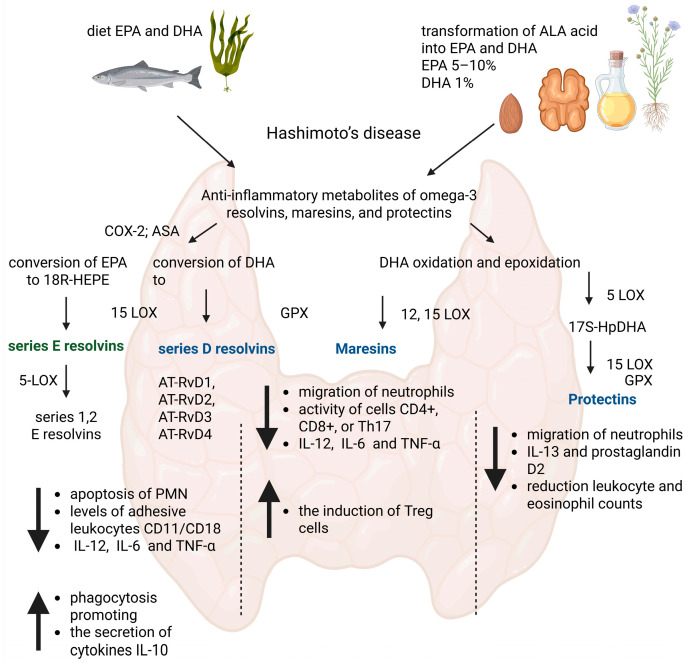
The contribution of anti-inflammatory mediators EPA and DHA in the suppression of the inflammatory response in Hashimoto’s disease. Created with BioRender.com/app.biorender.com.

Another derivative of omega-3 fatty acids with anti-inflammatory properties is protectin (PD1), also known as neuroprotectin (NPD1) due to its production in the nervous system. DHA serves as a precursor for its synthesis, similar to maresins. Initially, it is converted to 17S-HpDHA, a reaction involving 15-LOX. This intermediate product then undergoes epoxidation, leading to the formation of 16(17)-epoxydocosatriene, which, after enzymatic hydrolysis, yields the final product, PD1 [20]. Like maresins, protectin exhibits its anti-inflammatory action by reducing neutrophil migration, among other mechanisms. The presence of PD1 also results in a decreased amount of interleukin-13 (IL-13) and prostaglandin D2 (PGD2), both belonging to the group of pro-inflammatory mediators. Furthermore, studies have observed that the emergence of PD1 at inflammation sites leads to a reduction in leukocyte and eosinophil counts [21]. The contribution of anti-inflammatory mediators EPA and DHA in the suppression of the inflammatory response in Hashimoto’s disease is presented in Figure 1.

The objective of this work was to examine the impact of EPA and DHA fatty acids and their derivatives on the inflammatory state in Hashimoto’s disease. Investigating the correlation between the concentration of fatty acids and their derivatives in the body and the biochemical parameters of patients with Hashimoto’s could outline new therapeutic pathways for patients.

## 2. Materials and Methods

### 2.1. Research Methods and Tools

The study was conducted on a group of 33 female patients with confirmed Hashimoto’s disease, averaging a age of 37.58 ± 8.41 kg. Qualification for the study was based on an ultrasound image typical for cAITD (ALOKA Prosound Alpha-7 device, linear probe UST-5411 4.4–13.3 MHz, Tokyo, Japan) and an elevated level of anti-TPO antibodies (>34 IU/mL) and/or anti-TG (>115 IU/mL) in blood serum. Thyroid hormone status was determined based on the measurement of TSH (0.270–4.200 µIU/mL), fT4 (0.93–1.70 ng/dl), and fT3 (2.00–4.40 pg/mL). Fasting blood samples were drawn into polypropylene tubes containing EDTA. Using a refrigerated centrifuge, blood samples were spun for 10 min at 3000 rpm. The plasma was separated and collected into Eppendorf tubes and stored at −80 °C until analysis. In all patients, serum concentrations of TSH, fT3, fT4, anti-TPO, and anti-TG2 antibodies were determined using the electrochemiluminescence method (ECLIA) on a Roche Cobas model 6000 module 601 device (Indianapolis, IN, USA).

All patients received nutritional advice after the tests. The dietician analyzed the way of eating based on a food diary covering the three days before the control. Dietary interviews were assessed using the Diet 6D diet program recommended by the Institute of Food and Nutrition. The consumption of EPA and DHA fatty acids was low and amounted to appropriately 97.17 ± 267 mg and 21.68 ± 73.01 mg. The main source was occasionally consumed fish (salmon, trout, herring, pollock, cod).

From the obtained plasma, extraction was performed using RP-18 SPE (Agilent Technologies, Cheadle, UK) solid-phase extraction columns for 17-HEPE (cat. no. 3284), 17-HDHA (cat. no. 3365), 10(S)17(R)DiDHA (Protectin DX) (cat. no. 10008128), Maresin1 (cat. no. 11210), Rev D1 (cat. no. 10012554), and Rev E1 (cat. no. 10007848). To carry out eicosanoid extraction, 0.5 mL of plasma was added to 1 mL of acetonitrile to precipitate the protein, and 50 µL of internal standard (1 µg/mL) was added. Samples were incubated at −20 °C for 15 min and then centrifuged for 10 min at 10,000 rpm using a refrigerated centrifuge (Eppendorf, Centrifuge 5804R, Macquarie Park, NSW, Australia). The resulting supernatants were transferred to other tubes, and 4.5 mL of 1 mM HCl was added to each. Each tube was then added with 30–50 µL of 1 M HCl to adjust the pH to 3. The columns were then activated by sequentially washing with 3 mL of 100% acetonitrile and 3 mL of a 20% aqueous solution of acetonitrile. The samples were washed with a 20% acetonitrile solution. Eicosanoids were then eluted with 1.5 mL of a methanol and ethyl acetate mixture (1/1 *v*/*v*), dried under vacuum, and dissolved in a 60% methanol solution with 0.1% acetic acid. The samples were then analyzed using an Agilent Technologies 1260 HPLC, consisting of a degasser (model G1379B), binary pump (model G1312), column oven (model G1316A), and diode array detector (model G1315CDADVL+) [22]. The injection volume of the sample was 60 µL. The DAD detector monitored peaks by absorption at 235 nm for 17-HDHA, 280 nm for Rev E1, Protectin DX, Maresin1, and 302 nm for RevD1. Absorption spectra were analyzed to confirm the identification of analytes. The quantitative analysis was conducted using ChemStation software (Agilent Technologies, Cheadle, UK) [22].

### 2.2. Statistical Analysis

Statistical analysis was performed using StatView software (StatView 5.0, Cary, NC, USA). Using the Shapiro–Wilk test, it was verified that the collected results from the subjects had a distribution other than normal. Then, the data were analyzed using Spearman correlation. The results were considered statistically significant at *p* > 0.05.

## 3. Results

### Study Group

The youngest participant in the study was 22 years old, while the oldest was 49 years old (average = 37.6, SD = 8.406). The average body mass index (BMI) of the patients was 25.16 ± 4.13. All the average anthropometric and biochemical parameters, along with the standard deviation, are presented in Table 1. The thyroid-stimulating hormone (TSH) values of the examined patients ranged from 0.388 µLU/mL to 13.92 µLU/mL (3.27 ± 2.90). The average values of free triiodothyronine (fT3) of the subjects are 2.90 ± 0.470 pg/mL, and free thyroxine (fT4) is 1.26 ± 2.1 ng/dL, as shown in Table 1. Other inflammatory parameters (CRP, PLT, WBC) were not significantly elevated (Table 1).

The average content of eicosanoid acid in the examined patients was 4.39 ± 5.18. In the case of docosahexaenoic acid, the average was 6.75 ± 4.11. The content of Resolvina E1 ranged from 0.198 to 2.358; in the case of Resolvina D1 it was from 0.025 to 0.332. The mean values of Maresin1, 10S17RdiHDHA (Protectin DX), 18RS HEPE (18-hydro(peroxy)-eicosapentaenoic acid), and 17RS DHA were 1.26 ± 2.84, 0.25 ± 0.11, 0.38 ± 0.28, and 3.39 ± 1.95, respectively. All means are presented in the table below (Table 2).

No correlation was found between the levels of EPA and DHA acids and anthropometric and biochemical parameters. However, individual correlations were observed between derivatives and parameters. Statistical analysis showed positive average correlations between Resolvin D1 and body mass, %fat content, and fat mass (Table 3). Percent body fat content was also correlated with another resolvin (Resolvin E1). A certain relationship is also observed with maresin, which confirms the participation of EPA and DHA derivatives in the reduction in inflammation caused by the increase in adipose tissue (Table 3).

The analysis revealed a strong correlation between the level of 10S17R DiHDHA and C-reactive protein (CRP) and between 18-hydro(peroxy)-eicosapentaenoic acid (18RS HEPE) and CRP in the studied patients (Table 4). An average strength of correlation was also observed between the level of Maresin 1 and the level of fT4. A negative correlation of weak strength was noted between the level of 17RS HDHA and TSH in patients (Table 4). All data obtained during the statistical analysis are presented in the tables below (Table 3 and Table 4).

Significant results of the correlation analysis are additionally presented in graphical form (Supplement Figures S1–S6).

## 4. Discussion

As evidence has accumulated that specific dietary patterns may contribute to or modulate autoimmunity, a recent review examined the effects of *n*-3 polyunsaturated fatty acids (PUFAs), polyphenols, and fiber on rheumatic diseases (rheumatic arthritis and systemic lupus erythematosus) and thyroid diseases (such as Hashimoto’s thyroiditis) [23]. It was concluded that a healthy diet combined with physical activity is also useful in preventing weight gain and obesity, which are commonly associated with an increased risk of thyroid autoimmunity [24].

Omega-3 fatty acids and their metabolites are subjects of numerous studies regarding their role in aiding the treatment of diseases accompanied by inflammation. Omega-3s have shown therapeutic potential in autoimmune diseases like type 1 diabetes [22], rheumatoid arthritis [25], and systemic lupus erythematosus [26], attributed to their anti-inflammatory and regulatory properties. Gkiouras et al. investigated the effect of omega-3 supplementation on the anti-inflammatory activity and absorption of nonsteroidal anti-inflammatory drugs in chronic rheumatoid arthritis (RA) [27]. The certainty of the evidence was mostly low quality, but it was concluded that *n*-3 ONS in RA may have limited clinical benefit. Two systematic studies and meta-analyses noted some health benefits of omega-3 fatty acid (FA) supplementation. This included effects on inflammatory gene expression (IGE) in multiple sclerosis (MS) and the potential to lower markers of disease activity and absorption of nonsteroidal anti-inflammatory drugs in rheumatoid arthritis (RA) [28]. Studies on the effect of omega-3 in autoimmune thyroiditis are very scarce.

Promising preliminary findings regarding the association of the EPA derivative, specifically RvE1, with thyroid autoimmunity in Hashimoto’s thyroiditis were obtained in a study by J. Song et al. [29]. This study, which involved 30 untreated Hashimoto’s patients and 27 control patients matched by gender and age, revealed a significantly lower RvE1 level in Hashimoto’s patients. The study also reported a negative correlation of RvE1 with T3 and FT3 [30,31]. Although our own studies did not show such a relationship, inflammation with the participation of CRP was correlated with higher levels of D and E resolvins. In addition, our patients had more advanced disease because all of them were taking levothyroxine. Research into the association of fish consumption, with varying omega-3 content, and the concentration of ATPO and ATG antibodies was conducted among pregnant and postnatal women regularly consuming different fish species [32,33]. This enabled the estimation of the amount of omega-3s delivered through diet. Results indicate that women regularly consuming fatty fish, rich in EPA and DHA, had the lowest levels of anti-thyroid antibodies and, consequently, the lowest rate of postpartum thyroiditis [34]. This was confirmed by a subsequent expanded study involving 412 pregnant women [35]. Our study did not establish a correlation between the levels of antibodies and omega-3 but their derivatives were correlated with inflammation. It is likely that the physiological state and increased resolvin demand in the third trimester of pregnancy could be significant [36,37]. In another study, supplementation with omega-3 acids demonstrated a role in reducing inflammation through RvE1 in an animal asthma model. RvE1 mitigated airway reactivity and inflammation in asthmatic mice. Such results were only obtained in mice treated with RvE1 [37,38]. Supplementation with omega-3 acids alone, although they are precursors for resolvins, did not produce significant results [38]. One of the functions of anti-inflammatory mediators is the removal of apoptotic cells by phagocytes, i.e., efferocytosis. An improvement in this function was observed in a study conducted in a mouse model of severe plastic anemia that was treated with exogenous RvE1 [39]. Statistical correlations between CRP levels and RvE1, RvD1, 10S17R DiHDHA, and 18RS HEPE also suggest the therapeutic potential of omega-3s, especially their derivatives, in Hashimoto’s disease progression. The noted correlation implies that higher CRP levels among studied patients were associated with higher levels of the aforementioned EPA and DHA metabolites, counteracting inflammation. C-reactive protein (CRP) is an indicator of the acute phase of ongoing inflammation in the body [40]. Knowing the action mechanism of omega-3-derived mediators, this result can be interpreted to mean that as CRP increases and the acute phase of inflammation intensifies, the level of omega-3 derivatives rises to counteract it. These findings could influence the approach to treating HT and potentially improve the quality of life for patients. However, to validate this theory, it would be essential to conduct extensive studies over varied time periods and compare the results with those following the application of a healthy, well-balanced diet enriched with omega-3s. The statistically significant correlation of RvD1 with anthropometric measurements, such as fatty tissue mass, is also noteworthy, as increased fatty tissue mass is associated with chronic inflammation [41]. Studies have shown that administering anti-inflammatory mediators, especially RvD1, suppressed inflammation occurring in fatty tissue, evidenced by the reduction in pro-inflammatory adipokines like TNF-α, IL-6, and IL-1β [42].

### Study Limitations

The selection of women with Hashimoto’s disease in order to collect a homogeneous study group resulted in limitations in the number of patients. Due to the small sample size, it is possible that some relationships may not reach statistical significance even if true effects are present. Therefore, the conducted observations should be expanded in the future to a larger study group, taking into account gender and various thyroid diseases. Characteristics of the patient group should additionally include thyroid volume, echography patterns, serum levels of Tg (thyroglobulin), and urinary iodine secretion for the clinical information. More sensitive markers of chronic inflammation other than CRP and morphology blood should be used in the study. Unfortunately, at the study design stage they were not taken into account.

## 5. Conclusions

The chronic inflammatory state in Hashimoto’s thyroiditis (HT) is associated with intensified synthesis of anti-inflammatory mediators EPA and DHA to quell it. Anthropometric parameters dependent on fatty tissue are associated with an intensified inflammatory state and the extinguishing effect mediated by Resolvin D1, derived from DHA. DHA derivatives (maresin 1 and protectin 1) appear to be associated with the levels of fT4 and TSH, which may be associated with more advanced inflammation.

## Figures and Tables

**Table 1:** Characteristics of anthropometric parameters of the study group of women.

Parameter	Avg ± SD
Age [year]	37.58 ± 8.41
Height [cm]	167.32 ± 5.17
Body weight [kg]	70.59 ± 12.90
BMI [kg/m^2^]	25.16 ± 4.13
Fat tissue mass [kg]	25.181 ± 8.97
% body fat content	34.89 ± 6.96
ATPO [IU/mL]	197.72 ± 141.45
ATG [IU/mL]	326.2 ± 581.1
TSH [µLU/mL]	3.27 ± 2.91
fT3 [pg/mL]	2.90 ± 0.47
fT4 [ng/dL]	1.26 ± 0.21
CRP [mg/L]	2.24 ± 1.46
Blood platelets (PLT) [tys/mm^3^]	245.5 ± 50.79
Leukocytes (WBC) [tys/mm^3^]	5.8 ± 1.51
Neutrophils [tys/µL]	3.04 ± 1.18
Lymphocytes [tys/µL]	2.01 ± 0.41
Monocytes [tys/µL]	0.53 ± 0.14
Eosinophils [tys/µL]	0.17 ± 0.11
Basophils [tys/µL]	0.03 ± 0.01

BMI—body mass index; ATPO—thyroid peroxidase antibodies; ATG—antithyroglobulin antibodies; TSH—thyroid-stimulating hormone; fT3—free triiodothyronine; fT4—free thyroxine; CRP—C-reactive protein.

**Table 2:** Average content of analyzed fatty acids and their derivatives.

Fatty Acids and Their Derivatives	Avg	SD
C20:5n3 EPA	4.390	5.179
C22:6n3 DHA	6.751	4.105
Resolvin E1	0.736	0.539
Resolvina D1	0.154	0.077
Maresina 1	1.258	2.838
10S17R DiHDHA	0.247	0.114
18RS HEPE	0.376	0.281
17RS HDHA	3.386	1.949

EPA—eicosapentaenoic; DHA—docosahexaenoic; 10S17R DiHDHA—Protectin DX; 18RS HEPE—18-hydro(peroxy)-eicosapentaenoic acid); 17RS HDHA (dha acid derivative).

**Table 3:** Correlations of fatty acids and their derivatives with anthropometric parameters.

**EPA**	**R**	***p***
Age at study entry	0.24	0.248
Growth	0.085	0.6304
Body weight	0.043	0.8091
BMI	0.03	0.168
Fat tissue mass	0.032	0.8552
% body fat content	0.038	0.8301
**DHA**	**R**	***p***
Age at study entry	0.115	0.5154
Growth	−0.294	0.0259
Body weight	−0.113	0.5237
BMI	−0.02	0.9093
Fat tissue mass	−0.6	0.7336
% body fat content	−0.055	0.7536
**Resolvin E1**	**R**	***p***
Age at study entry	0.26	0.882
Growth	0.024	0.891
Body weight	0.293	0.098
BMI	0.286	0.1061
Fat tissue mass	0.29	0.1004
% body fat content	**0.349**	**0.0486**
**Resolvin D1**	**R**	***p***
Age at study entry	−0.55	0.754
Growth	0.211	0.2334
Body weight	**0.365**	**0.0388**
BMI	**0.332**	**0.0601**
Fat tissue mass	**0.36**	**0.0418**
% body fat content	**0.363**	**0.0402**
**10S17R DiHDHA**	**R**	***p***
Age at study entry	0.042	0.8124
Growth	0.17	0.3354
Body weight	0.201	0.2548
BMI	0.16	0.3649
Fat tissue mass	0.007	0.9683
% body fat content	0.012	0.9442
**Maresin 1**	**R**	***p***
Age at study entry	0.165	0.3503
Growth	0.06	0.7329
Body weight	0.146	0.4089
BMI	0.133	0.4532
Fat tissue mass	0.324	0.669
% body fat content	0.32	0.0704
**18RS HEPE**	**R**	***p***
Age at study entry	−0.085	0.6317
Growth	0.135	0.4458
Body weight	0.103	0.5619
BMI	0.04	0.8194
Fat tissue mass	0.14	0.4277
% body fat content	0.192	9.2766
**17RS HDHA**	**R**	***p***
Age at study entry	0.086	0.627
Growth	0.219	0.2159
Body weight	0.018	0.9168
BMI	−0.04	0.8231
Fat tissue mass	0.124	0.4813
% body fat content	0.201	0.2554

BMI—body mass index; DHA—docosahexaenoic; EPA—eicosapentaenoic; 10S17R DiHDHA—Protectin DX; 18RS HEPE—18-hydro(peroxy)-eicosapentaenoic acid); 17RS HDHA—(dha acid derivative). Bold-statistically significant.

**Table 4:** Correlations of fatty acids and their derivatives with biochemical parameters.

**EPA**	**R**	***p***
ATPO	−0.196	0.2685
ATG	−0.081	0.6483
TSH	0.058	0.7411
fT3	−0.117	0.5084
fT4	−0.078	0.6592
CRP	0.242	0.1716
**DHA**	**R**	***p***
ATPO	−0.031	0.8601
ATG	−0.009	0.9574
TSH	−0.071	0.6875
fT3	0.257	0.1466
fT4	0.162	0.3589
CRP	−0.045	0.8
**RvE1**	**R**	***p***
ATPO	−0.149	0.3999
ATG	−0.141	0.4258
TSH	−0.207	0.2405
fT3	0.151	0.3925
fT4	0.055	0.7554
CRP	**0.395**	**0.0254**
**RvD1**	**R**	***p***
ATPO	−0.039	0.823
ATG	−0.071	0.6875
TSH	0.042	0.8135
fT3	0.079	0.6561
fT4	0.079	0.6534
CRP	**0.336**	**0.057**
**10S17R DiHDHA**	**R**	***p***
ATPO	−0.067	0.7043
ATG	0.034	0.8482
TSH	−0.028	0.8575
fT3	0.057	0.7454
fT4	−0.085	0.6321
CRP	**0.523**	**0.0031**
**Maresina 1**	**R**	***p***
ATPO	−0.072	0.6854
ATG	−0.017	0.9228
TSH	−0.256	0.1474
fT3	0.068	0.7015
fT4	**0.355**	**0.0447**
CRP	0.023	0.8962
**18RS HEPE**	**R**	***p***
ATPO	−0.228	0.1981
ATG	−0.205	0.2459
TSH	−0.098	0.5799
fT3	0.004	0.9842
fT4	0.15	0.397
CRP	**0.494**	**0.0052**
**17RS HDHA**	**R**	***p***
ATPO	−0.202	0.2541
ATG	−0.024	0.8913
TSH	**−0.347**	**0.0499**
fT3	0.131	0.4572
fT4	0.309	0.0801
CRP	0.179	0.3113

ATPO—thyroid peroxidase antibodies; ATG—antithyroglobulin antibodies; TSH—thyroid-stimulating hormone; fT3—free triiodothyronine; fT4—free thyroxine; CRP—C-reactive protein.

## Data Availability

The original contributions presented in this study are included in the article. Further inquiries can be directed to the corresponding author.

## Supplementary Materials

The following supporting information can be downloaded at https://www.mdpi.com/article/10.3390/nu17101715/s1. Figure S1. Correlation between Resolvin D1 and CRP. Figure S2. Correlation between Resolvin E1 and CRP. Figure S3. Correlation between 18RS HEPE and CRP. Figure S4. Correlation between 10S17R DiHDHA and CRP. Figure S5. Correlation between Maresina 1 and FT4. Figure S6. Correlation between 17RS HDHA and TSH.
