# Supplementary material for: The Significance and Process of Inflammation Involving Eicosapentaenoic and Docosahexaenoic Derivatives in Hashimoto’s Disease
Source: Nutrients. 2025 May 19;17(10):1715. doi: 10.3390/nu17101715 (PMC12113837; doi:10.3390/nu17101715)
Supplement: Supplementary file 1 [file nutrients-17-01715-s001.zip › nutrients-3635766-supplementary.pdf]

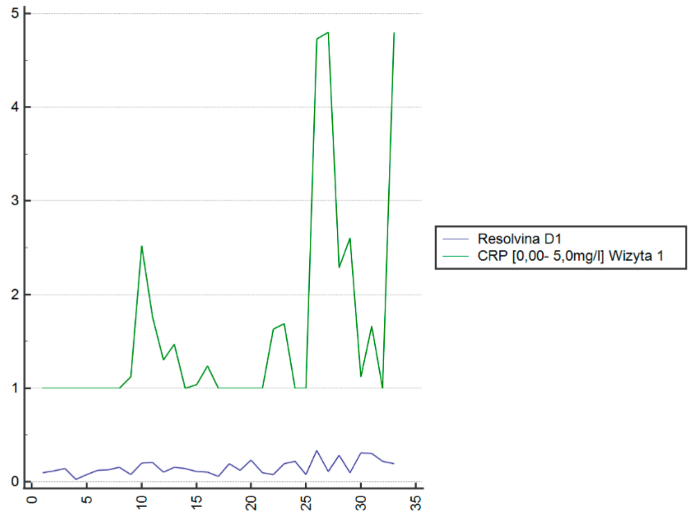

Figure S1. Correlation between Resolvin D1 and CRP.

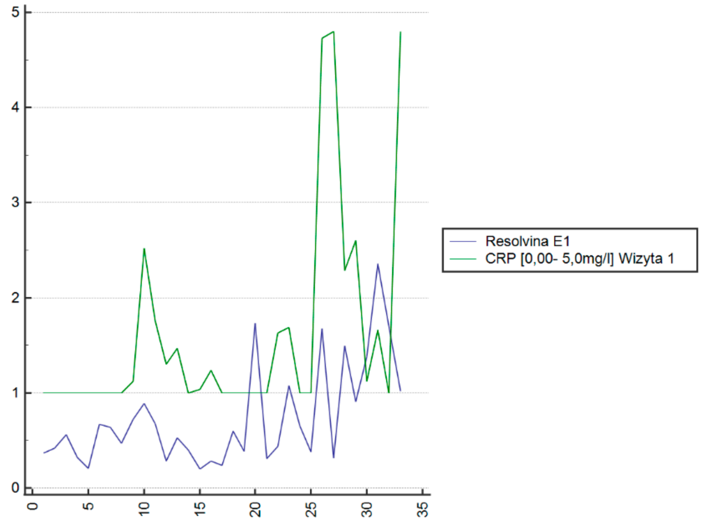

Figure S2. Correlation between Resolvin E1 and CRP.

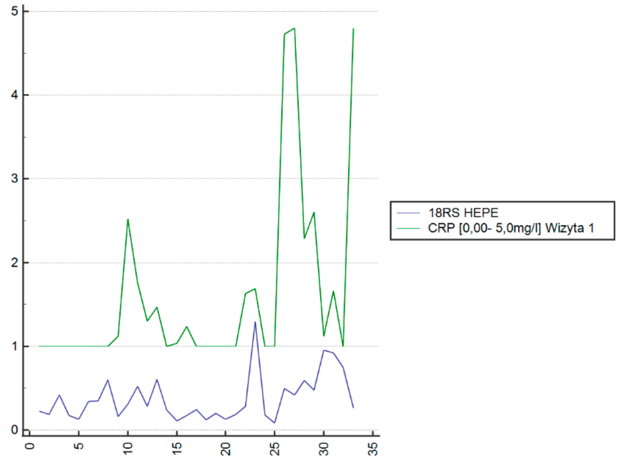

Figure S3. Correlation between 18RS HEPE and CRP.

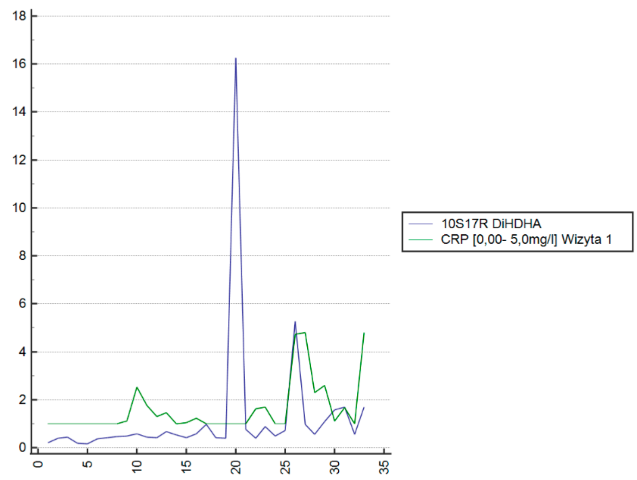

Figure S4. Correlation between 10S17R DiHDHA and CRP.

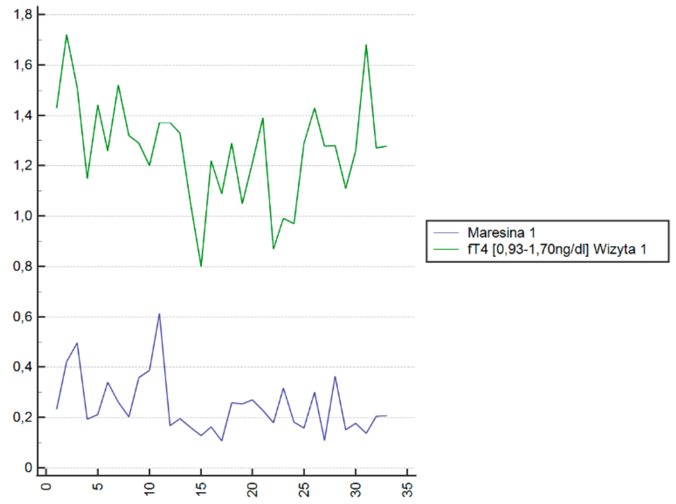

Figure S5. Correlation between Maresina 1 and FT4.

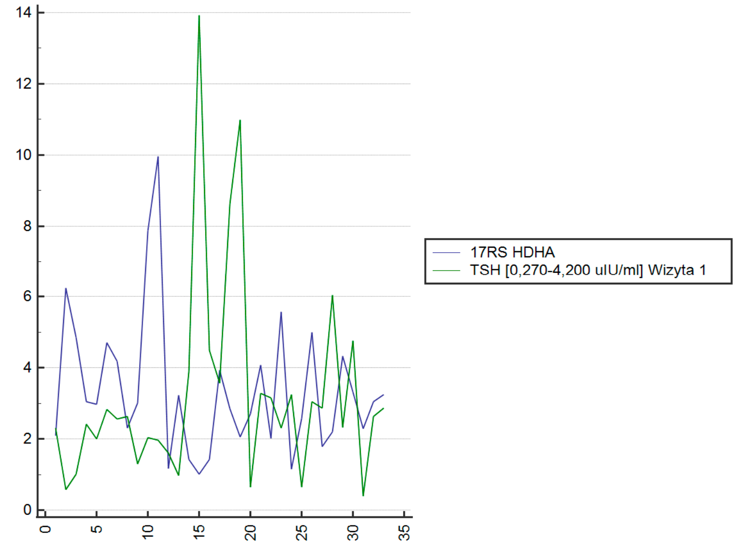

Figure S6. Correlation between 17RS HDHA and TSH.
